# Editorial: Enhanced human modeling in robotics for socially-aware place navigation

**DOI:** 10.3389/frobt.2024.1348022

**Published:** 2024-03-01

**Authors:** Konstantinos A. Tsintotas, Ioannis Kansizoglou, Katerina Pastra, Yiannis Aloimonos, Antonios Gasteratos, Giorgios Ch. Sirakoulis, Giulio Sandini

**Affiliations:** ^1^ Democritus University of Thrace, Komotini, Greece; ^2^ Athena Research Center, Marousi, Greece; ^3^ University of Maryland, Baltimore, MD, United States; ^4^ Italian Institute of Technology (IIT), Genova, Liguria, Italy

**Keywords:** robotics, social navigation, AI, machine learning, language processing

## 1 Introduction

Autonomous and accurate navigation is a prerequisite for any intelligent system assigned to various missions. Yet, this task presents a higher complexity when a mobile robot navigates in an unfamiliar terrain, as it needs to move through the environment and construct a detailed map of its surroundings. At the same time, the system should estimate its pose and orientation during the incremental construction of its internal map (Tsintotas et al., 2022). This process is widely known as simultaneous localization and mapping (SLAM) and is paramount for effective and context-aware navigation. However, this challenge becomes even more intricate when robots work within human environments, as human-robot coexistence introduces variables such as human activities, intentions, and their impacts on the robot’s path (Keroglou et al., 2023). At the same time, the integration necessitates adherence to stringent safety and security requirements. Consequently, the robotic community tries to tackle these challenges through several techniques that collectively shape the field into a demanding, interdisciplinary pursuit known as socially aware navigation. This involves technical considerations and a deep understanding of the social dynamics between humans and robots, marking a crucial intersection of robotics, artificial intelligence, and human-computer interaction. Should we understand human activities, intentions, or social dynamics via intelligent pipelines, robots can navigate spaces shared with humans, fostering a harmonious coexistence, *e.g.*, healthcare, or assistive technologies to smart homes and public space. Last, socially aware robot navigation aims to bridge the gap between artificial intelligence and human interaction, paving the way for a more integrated and socially intelligent future.

## 2 Analysis of the Research Topic

**FIGURE 1:**
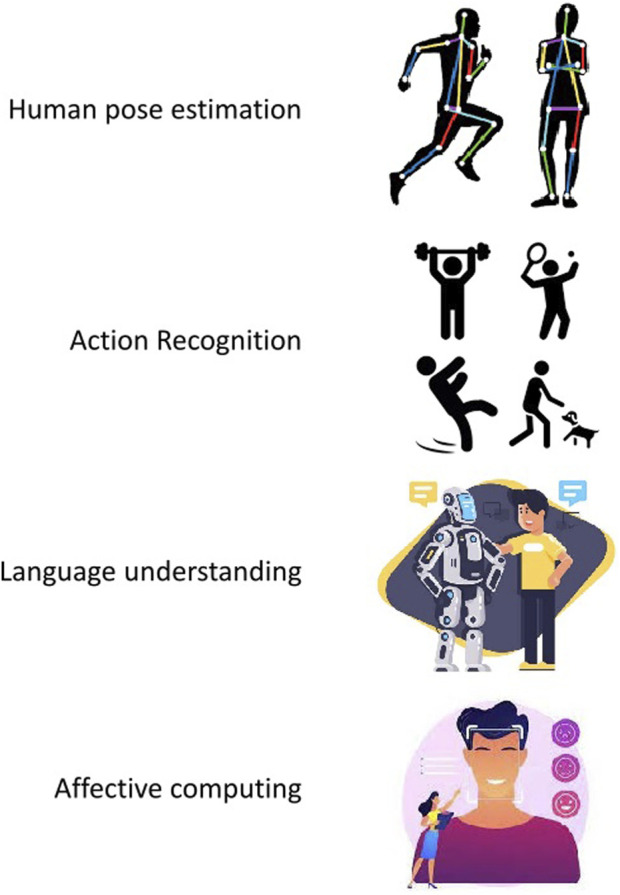
Socially aware place navigation dimensions. Human pose estimation is responsible for determining a person’s body’s spatial configuration, enabling robots to precisely interpret and respond to human movements. Human action recognition involves identifying and classifying specific movements or behaviors performed by a person or a group, and this way, an autonomous agent can understand and respond in various contexts. Next, language understanding concerns the capability to comprehend and interpret natural language input. Due to this fact, effective communication and collaboration between humans and robots can be reached. Last, affective computing focuses on developing techniques that can recognize and interpret human emotions, enhancing the ability of social robots to engage in emotionally intelligent interactions with users.

The paradigm of socially aware place navigation is situated within the intricate domain of human modeling, systematically examining various dimensions such as human pose estimation (Wei et al., 2022), action recognition (Charalampous et al., 2017; Dessalene et al., 2021), language understanding (Vatakis and Pastra, 2016), and affective computing (Kansizoglou et al., 2022) (see Figure 1). The first is the discernment of the spatial configuration of an individual’s body, a pivotal facet enabling a robotic system to comprehend humans’ physical presence and movements within its proximate environment (An et al., 2022). At the same time, action recognition further augments this comprehension by interpreting the activities in which individuals are engaged (Dessalene et al., 2023), thereby contributing to a nuanced understanding of the contextual environment (Moutsis et al., 2023). Language understanding, a fundamental component of this multifaceted paradigm, empowers the robot to discern verbal cues and commands (Pastra and Aloimonos, 2012), thereby facilitating seamless communication with human counterparts. At the same time, affective computing introduces an emotional dimension, endowing the robot to discern and appropriately respond to human emotions, enhancing its adaptability to intricate social contexts (Kansizoglou et al., 2019). Last, the amalgamation of these human-centric capacities within the purview of the navigation task epitomizes a sophisticated methodological approach, and consequently, such frameworks are poised to excel in scenarios characterized by adversity, dynamism, and heightened interactivity.

### 2.1 Contributing articles

Although user-centered approaches are essential to create a comfortable and safe human-robot interaction, they are still rare in industrial settings. Aiming to close this research gap, in Bernotat et al., two user studies with large heterogeneous samples were conducted. In particular, in User Study 1, the participants’ ideas about robot memory were explored, as well as what aspects of the robot’s movements were found positive, and what they would change. The effects of participants’ demographic backgrounds and attitudes were controlled for. Next, it is self-evident that even in such an elementary and minimal environment compared to the real world, home agents require guidance from dense reward functions to learn to carry out complex tasks. As task decomposition is an easy-to-use approach for introducing those dense rewards, in Petsanis et al., a method that can be used to improve training in embodied AI environments by harnessing the task decomposition capabilities of TextWord is presented. On the other hand, Karasoulas et al. examined how to detect the presence or absence of individuals indoors by analyzing the ambient air’s CO_2_ concentration using simple Markov Chain Models. While this study focused on employing 1-h window testing sets, there exists significant potential for accurately assessing occupancy profiles within shorter minute intervals. At last, the authors in Arapis et al. focus on localizing humans in the world and predicting the free space around them by incorporating other static and dynamic obstacles. Their research is based on a multitasking learning strategy to handle both tasks, achieving this goal with minimal computational demands when employed in difficult industrial environments, such as human instances at a close distance or the limits of the field of view of the capturing sensor.

## 3 Discussion and conclusion

Overall, the main objective of a human-aware navigation pipeline is to facilitate human-robot coexistence in a shared environment. Such a scenario requires the efficient parallel realization of each member’s goals without needless external interceptions or delays and the successful completion of specific everyday tasks. On top of that, the robotic agent is expected to inspire a sense of trust and friendliness in humans, mainly realized when the agent operates concisely, adaptively, transparently, and naturally. Thus, robot navigation techniques shall employ enhanced human understanding and modeling techniques, capturing those features that mainly affect the efficiency of the task. As a result, it becomes increasingly vital to develop robust, lightweight action and affect estimation solutions based on robotics sensory data and capacities, like active vision (Aloimonos et al., 1988). Finally, computational efficiency and real-time operation capacities always limit the introduced solutions.
